# The effect of novel nitrogen-rich plasma polymer coatings on the phenotypic profile of notochordal cells

**DOI:** 10.1186/1475-925X-6-33

**Published:** 2007-09-06

**Authors:** Fackson Mwale, Hong Tian Wang, Alain Petit, Pierre-Luc Girard-Lauriault, Christopher J Hunter, Jean A Ouellet, Michael R Wertheimer, John Antoniou

**Affiliations:** 1 Division of Orthopaedic Surgery, McGill University, and Lady Davis Institute for Medical Research, 3755, Chemin de la Cote Ste-Catherine, Montreal, QC H3T 1E2, Canada; 2 Department of Engineering Physics, Ecole Polytechnique, Montréal, QC H3T 1J4, Canada; 3 McCaig Centre for Joint Injury and Arthritis Research, Department of Civil Engineering, Department of Cell Biology and Anatomy, University of Calgary, Calgary, AB T2N 1N4, Canada; 4 Montreal Children Hospital, 2300 Tupper Street, Room C-521, Montréal, QC H3H 1P3, Canada

## Abstract

**Background:**

The loss of the notochordal cells from the nucleus pulposus is associated with ageing and disc degeneration. However, understanding the mechanisms responsible for the loss of these cells has been hampered in part due to the difficulty of culturing and maintaining their phenotype. Furthermore, little is known about the influence of the substratum on the molecular markers of notochordal cells.

**Methods:**

Notochordal cells were isolated from lumbar spine of non-chondrodystrophoid dogs and cultured on N-rich plasma polymer layers, so-called "PPE:N" (N-doped plasma-polymerised ethylene, containing up to 36% [N]) surfaces, for 3, 7 or 14 days. Gene expression of vimentin (VIM), pleiotrophin (PTN), matrix Gla protein (MGP), cartilage oligomeric matrix protein (COMP), keratin 18 (KRT 18), aggrecan (AGG), collagen type 1 (COL1A2), collagen type 2 (COL2A1) was analyzed through semi-quantitative reverse transcription-polymerase chain reaction (RT-PCR).

**Results:**

Notochordal cells were maintained in culture on PPE:N for up to 14 days with no loss in cell viability. Except for VIM, gene expression varied depending on the culture periods and [N] concentration of the substratum. Generally, PPE:N surfaces altered gene expression significantly when cells were cultured for 3 or 7 days.

**Conclusion:**

The present study has shown that notochordal cells from dogs can attach to and grow on PPE:N surfaces. Analysis of the expression of different genes in these cells cultured on different N-functionalized surfaces indicates that cellular behaviour is gene-specific and time-dependent. Further studies are required to better understand the roles of specific surface functionalities on receptor sites, and their effects on cellular phenotypes.

## Background

Diseases of the spine that afflict the elderly population involve defects in the intervertebral disc (IVD). Low back pain is an insidious disorder that, by age 70, affects about 60% of the population. Although the aetiology of low back pain is often unclear, it is believed that IVD degeneration plays a major role [1]. Surgical treatments of lumber spine disorders, including degenerative disc diseases, consist of disc excision and vertebral fusion [2,3]. Although surgical procedures produce a good short-term clinical result in relief of pain, they alter the biomechanics of the spine, leading to further degeneration of surrounding tissue and discs at adjacent levels. Failure rates for lumbar fusions are 20% to 40% after five years [4]. As clinical and radiological evidence suggest that spinal fusion leads to accelerated degeneration of adjacent motion segments and early failure, alternative treatments are needed. Indeed, present management of disc pathology has been focused on symptoms associated with degeneration and much less study has been devoted to disc regeneration.

IVDs allow bending and twisting of the spine whilst resisting compression from gravity and muscle action. They are composite structures of the peripheral collagen-rich annulus fibrosus (AF) surrounding the proteoglycan-rich central nucleus pulposus (NP). Their development is complex and involves several different connective tissue types [5]. Discs are characterized by their abundant extracellular matrix (ECM) and low cell density, coupled with an absence of blood vessels, lymphatic system, and nerves in all but the most peripheral annular layers [5]. The discs are thought to resist compressive forces by their high content of the proteoglycan aggrecan. Many aggrecan molecules can bind to a single hyaluronate chain, producing large proteoglycan aggregates, with each interaction being stabilized by the further interaction of a link protein. These proteoglycan aggregates induce a high swelling pressure in the NP that is balanced by tensile forces produced in the collagen network of the AF. With aging, IVD proteoglycans undergo marked changes in their metabolism and composition, leading to reduction in aggrecan content due to decreased proteoglycan synthetic activity or increased degradation [6].

The IVD of some species, including humans, contains residual cells from the embryonic notochord. These cells form large three-dimensional clusters in the young, healthy disc but may eventually be lost, either through apoptosis or terminal differentiation, and are replaced by chondrocyte-like cells during aging and degeneration [7]. The reduction in their numbers in the NP after birth in humans and in the chondrodystrophoid dog correlates with early degenerative changes in the disc and with a concomitant reduction in proteoglycan content, increased collagen, and loss of water content [7]. However, little is known about the basic mechanism of this accelerated degeneration with ageing. It is known that NP cells co-cultured with notochordal cells exhibit an increased proteoglycan synthesis as a result of soluble factor(s) produced by notochordal cells [8]. It is therefore, possible that notochordal cells can be used in tissue engineering of the IVD.

We recently fabricated and used novel bioactive synthetic polymer coatings, named nitrogen(N)-doped plasma-polymerised ethylene (PPE:N) [9]. These new biocompatible substrates, with desirable chemically-bound N-functionalities, can help to control cellular events during cell or tissue culture. Thus, studying the interactions of cells with these new material surfaces, particularly the way PPE:N of differing N concentrations, [N], influences various cellular responses, is very important. Some of the questions that now arise are the following: can notochordal cells adhere to and develop on our bioactive synthetic PPE:N surfaces? Will gene expression be affected by these substrates? To answer these questions, notochordal cells were isolated from dogs, cultured on different PPE:N surfaces, and the expression of vimentin (VIM), pleiotrophin heparin binding factor (PTN), matrix gla protein (MGP), cartilage oligomeric matrix protein (COMP), and keratin (KRT) genes analyzed. These genes are expressed differently in NP cells than in AF cells of rats [10], suggesting that these proteins may have important functions in the NP and that their genes may be used as markers. We also studied the expression of aggrecan and types I and II collagens because they are important constituents of the ECM in IVD tissue [11]. To the best of our knowledge, this is the first time that the interactions of notochordal cells with various materials' surfaces have been examined and reported.

## Methods

### Polymers with modified surfaces

Synthetic polymers are used extensively as biomaterials [12,13] in a wide variety of applications such as delivery systems for drugs [14-17], proteins [18], and genes [17], recognition systems [19,20], tissue engineering [21-25], and cell culture [26-29]. In their pristine state, however, these materials are characterized by low surface energies which, in turn, result in poor wettability by water and physiological fluids (which comprise mostly water). This further leads to poor adhesion, for example, of living cells. However, this drawback of synthetic polymers can be readily overcome by chemically modifying the polymers' surfaces, without affecting their many desirable bulk properties [9,27-29]. In other words, new chemical functionalities can be created at the surface, or within a very shallow (nanometer thin) surface-near layer of the material. One of the most convenient and economical ways of accomplishing this is to use low-temperature plasmas, either low-pressure glow discharges or atmospheric-pressure ("corona") discharges [30-32]. Commercial tissue culture substrates are typically polystyrene (PS) that has been modified in such a way: the ones used here (see further below) were found to contain 18% of oxygen at the surface, which is chemically-bound in the form of various polar moieties that enhance wettability and cell adhesion. On another commercial tissue culture substrate, Primaria^®^, we have found 6% and 15% of chemically bound nitrogen (N) and oxygen (O), respectively. However, these plasma-modified polymers all manifest an undesirable phenomenon known as ageing (or hydrophobic recovery), a gradual loss of wettability over time [30]. For this and other reasons, we use thin PPE:N coatings, described in the following section.

### Deposition of PPE:N

Nitrogen (N)-rich plasma-polymerised ethylene (PPE:N) coatings have the advantages that the concentration of bound N, [N], can be varied over a very broad range (17% < [N] < 36%), and that this value at the surface does not change with time. In other words, no undesirable "ageing" effects are observed [9,30]. The methods employed for depositing PPE:N coatings were described earlier by Girard-Lauriault *et al*. [9]. For the experiments reported here, PPE:N films were deposited on biaxially oriented polypropylene (BOPP); the characteristics of this 50 μm- thick isotactic polymer film, graciously provided by 3 M Company, have also been described elsewhere [33]. As explained earlier [9], changing the flow rate of the ethylene gas precursor, F_C2H4_, in the mixture with pure nitrogen (N_2_) gas from the lowest value of 5 standard cubic centimeters per minute (5 sccm) to higher values gradually reduces [N]. Thus, for F_C2H4 _= 5 sccm, [N] ≈ 36%, while for the highest F_C2H4 _used here, 60 sccm, [N] ≈ 17 %, all other deposition conditions of course being maintained constant. In later parts of this text we shall refer to these different PPE:N coatings in terms of the F_C2H4 _values used in their preparation, namely 5, 10, 20 and 60 sccm, knowing that the resulting different [N] values can lead to significantly different cell responses [9]. Accordingly, as shown in Table 1, coatings are hereafter designated S5, S10, S20 and S60, in the order of increasing F_C2H4 _(decreasing [N]) values.

**Table 1:** Characteristics of PPE:N coatings and of PS control surfaces.

Sample designation	Nitrogen Flow Rate, F_N2 _(slm)	Ethylene Flow Rate, F_C2H4 _(sccm)	Elemental Concentrations (%)		
			
			Nitrogen, [N]	Oxygen, [O]	Carbon [C]
PS Control	N.A.	N.A.	0	18.0	82.0
S5	10	5	36.0	9.0	55.0
S10	10	10	29.5	7.0	63.5
S20	10	20	25.1	1.2	73.7
S60	10	60	18.0	5.0	77.0

The surface compositions of PPE:N films (Table 1) were determined by X-ray photoelectron spectroscopy (XPS) as previously described [9,33]; throughout this article, we will be referring to their surface elemental concentrations, [X], in terms of the elements that comprise PPE:N, in particular their total nitrogen content, [N]. However, more realistically, the substrates' effect on adhering cells is mediated by the concentrations of various chemical functionalities at the surface, for example amines, imines, nitriles, amides, acids and alcohols (bound oxygen is always incorporated in plasma polymer films due to the reaction of residual surface radicals with ambient air). Unfortunately, plasma polymers are difficult to characterize, on account of their random, highly cross-linked structure; the resulting peak broadening, therefore, greatly complicates quantitative, even qualitative, analysis by most spectroscopic methods. However, we do know that the primary amines account for 10 to 15 % of [N], and that nitriles (-C ≡ N) also constitute an important surface functionality [34].

### Cell isolation

Notochordal cells were isolated from canine lumbar spines using the method of Hunter *et al *[35], which was approved by University of Calgary's Animal Care Committee with the reference number M03126. Lumbar IVDs were collected from young, skeletally mature, mongrel dogs (age < 2 years, 20–25 kg) within two hours of euthanasia. The dogs were purpose-bred mongrels from a single breeder using primarily German shepherd and husky stock, and were therefore presumably non-chondrodystrophoid [36]. The discs were all categorized as stage 1 on the Bray and Burbidge scale [37], with a gelatinous nucleus pulposus, distinct nuclear-annular demarcation, and normal annular lamellae (approximately equivalent to grade I on the Thompson scale for human IVDs [38]). The lumbar spine was removed *en bloc *and transferred to a cell culture hood, where the IVDs were cut open and the nuclei pulposi were removed and transferred to phosphate-buffered saline (PBS). Following isolation, the nuclei pulposi were pooled and then digested using a modified version of the method described by Maldonado and Oegema [39]. Briefly, nucleus pulposus tissue was digested for 90 minutes in 0.4% w/v Pronase (Roche Applied Science, Laval, QC, Canada) and 5% fetal bovine serum (FBS) (Invitrogen, Burlington, ON, Canada), followed by an overnight digestion in 0.012% w/v collagenase type II (Sigma-Aldrich, Burlington, ON, Canada) and 5% FBS. The resulting digest was filtered through a sterile 70 μm nylon mesh filter and washed twice with PBS.

### Cell culture

**Figure 1:**
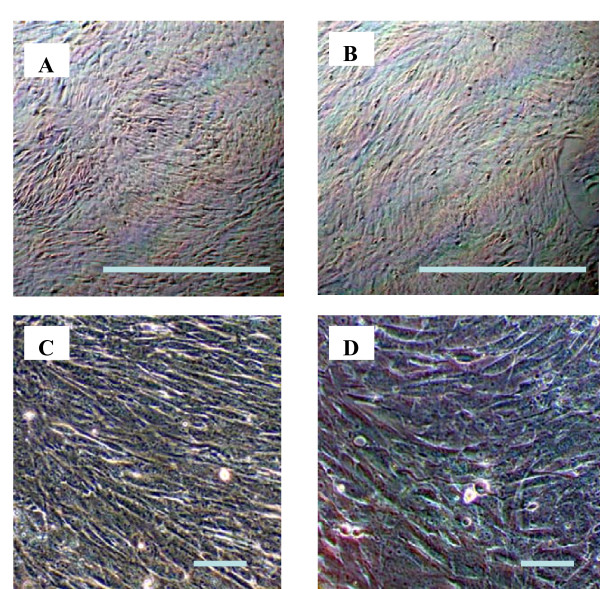
**Dog notochordal cells in culture**. Dog notochordal cells were cultured for 7 days on PS control (A, C) and S20 PPE:N (B, D) surfaces. Pictures were taken using an Axiovert 25 microscope (Carl Zeiss Canada, Kirkland, QC Canada). Similar results were obtained with the other PPE:N surfaces. The bars in A and B equals 250 μm while in C and D equals 50 μm.

Commercial polystyrene (PS) tissue culture dishes (100 × 20 mm) (Sarstedt, Montreal, QC, Canada) were used as controls (hereafter designated "PS control"). These commercial culture dishes are known to be plasma treated for improved hydrophilicity and cell adhesion, and our XPS measurements show that they possess a surface oxygen concentration, [O], of 18%. The notochordal cells were cultured on these PS control surfaces and on four different PPE:N coatings (about 100 mm × 100 mm), S5, S10, S20, and S60, in DMEM-high glucose, supplemented with 10% FBS, 100 U/ml penicillin, and 100 μg/ml streptomycin. At the beginning, 5 × 10^5 ^cells were cultured in every dish for 3-day gene expression analysis, 3 × 10^5 ^cells for 7-day analysis, and 2 × 10^5 ^cells for 14-day analysis. The medium was changed every 2 days. Inverted light microscopy pictures show that cell growth was similar on PPE:N as on PS control surfaces (Figure 1A and 1B). However, due to the difference in the size of these surfaces it is very difficult to compare the exact numbers of cells. At higher magnification (400X), the morphology of the cells appeared to be very similar (Figure 1C and 1D). It is important to note that at day 3, 7, and 14, cells were washed with PBS and recovered by adding 1 ml of TRIzol reagent (Invitrogen, Burlington ON, Canada) for gene expression analysis. For every culture condition, the experiments were repeated five (5) times.

### Total RNA isolation

Total RNA was extracted from notochordal cells by a modification of the method of Chomcynski and Sacchi [40] using TRIzol reagent (Invitrogen). The aqueous phase was precipitated in 1 volume of isopropanol. The resulting RNA pellet was air-dried and resuspended in 40 μl diethylpyrocarbonate-treated distilled water. RNA concentration and purity was assayed by measuring A_260 _and A_260_/A_280_.

### Reverse transcription (RT) and polymerase chain reaction (PCR)

The RT reactions for cDNA synthesis were performed using 1 μg total RNA isolated from the notochordal cells in a total volume of 20 μ1, containing 50 mM Tris-HCl (pH 8.3), 75 mM KCl, 3 mM MgCl_2_, 10 mM DTT, 50 μM each dATP, dGTP, dCTP and dTTP, and 200 units of Superscript II – RNAse H^-^reverse transcriptase (Invitrogen).

PCR was performed in a total volume of 25 μl containing 10 mM Tris-HCl (pH 8.3), 1.5 mM MgCl_2_, 0.4 mM of dATP, dGTP, dCTP, dTTP, 0.8 μM of each primer, 1 μl of RT mixture and 2.5 units of Taq DNA polymerase (Invitrogen) as previously described [27,28]. The 30 cycles of PCR included denaturation (94°C, 1 min), annealing (56°C, 45 sec) and extension (72°C, 40 sec). After agarose (2%) gel electrophoresis, PCR products were visualized by ethidium bromide staining and analyzed using a Bio-Rad VersaDoc image analysis system, equipped with a cooled 12 bit CCD camera (Bio-Rad, Mississauga ON, Canada). Glyceraldehyde-3-phosphate dehydrogenase (GAPDH) was used as housekeeping gene and served to normalize the results. To confirm the absence of chromosomal DNA contamination of RNA samples, PCR was also performed with RNA aliquots.

The primers were designed according to the dog gene sequences. However, because of problems we encountered in the gene bank with dog sequences, the bovine primers for GAPDH and AGG were used. The primer sequences are shown in Table 2 and they were synthesized by Invitrogen. All of these primers amplify a single product.

**Table 2:** Sequences of primers for RT-PCR

AGG	Forward: CAG AAC ATG CGC TCC AAT GA Reverse: CGT CAT AGG TTT CGT TGG TG	370 bp
COL 1A2	Forward: TGC AGT AAC TTC GTG CCT AG Reverse: AAT CCA TCC AGA CCA TTG TG	525 bp
COL 2A1	Forward: agt gct gtc cca tct gct ca Reverse : GCC TTC TCA TCA AAT CCT CCA	322 bp
COMP	Forward: aca gtg atg gag tgt gac gcd Reverse: GTT GCA CTC GTT GAC GTC GA	201 bp
GAPDH	Forward: TAT GAC CAC TGT CCA CGC CAT Reverse: AGT ATC GCT GTT GAA GTC GCA	349 bp
KRT 18	Forward: aga tcg agg ctc tca agg ag Reverse: GCC TTC AGA TTT CTC ATG GAG T	349 bp
MGP	Forward: tgc tcc ttc tct cca ttc tg Reverse: GCT TGA AGT CAT CAC AGG CT	222 bp
PTN	Forward: TGA CTG TGG AGA ATG GCA GTG Reverse: CCG TAT TCA GGT CAC ATT CT	293 bp
VIM	Forward: TGC AGG ATG AGA TTC AGA ACA Reverse: ACC GTC TTA ATC AGA AGC GT	246 bp

**AGG **– aggrecan,**COL1A2 **– collagen, type I, alpha 2, **COL2A1 **– collagen, type II, alpha1, **COMP **– cartilage oligomeric matrix protein, **GAPDH **– glyceraldehyde-3-phosphate dehydrogenase, **KRT18 **– keratin-18, **MGP **– matrix Gla protein, **PTN **– pleiotrophin, **VIM **– vimentin.

### Statistical analysis

Statistical significance was calculated using ANOVA followed by Fisher's PLSD comparison test using Statview (SAS Institute Inc., Cary, NC). Results were considered statistically significant at p < 0.05. Results are the mean ± standard error of 5 samples.

## Results

### Characteristics of PPE:N coatings

In an earlier article, we reported in considerable detail the methodology of depositing PPE:N coatings on BOPP and on other (polymeric or glass) substrates, as well as methods used for characterizing the resulting thin film materials, and the biological responses, mostly adhesion or non-adhesion of various cell types of interest to orthopaedics [9]. In order not to unduly repeat those earlier-published data, we have limited ourselves here to describing how [N] varies with F_C2H4_. As shown earlier [9], [N] values decrease in a non-linear monotonic manner from the maximum value, [N] ~36% for the lowest value of F_C2H4 _(5 sccm), to [N] ~18% for the highest F_C2H4 _value used, 60 sccm (Table 1).

### Expression of vimentin (VIM)

**Figure 2:**
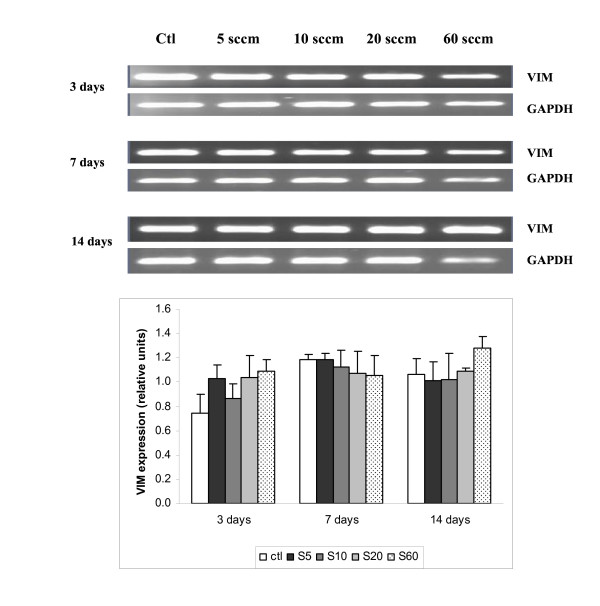
**VIM expression in dog notochordal cells cultured on five different substrates**. Dog notochordal cells were cultured for up to 14 days on PS control surface and four different PPE:N surfaces. GAPDH was used as housekeeping gene and served to normalize the results. Agarose gels are representative of 5 experiments. Quantitative results are the mean ± standard error of these five experiments. * p < 0.05 vs. PS control.

Since VIM mRNA has recently been discovered to be expressed at significant levels in the NP [10], we first explored whether this gene is also expressed by notochordal cells. Figure 2 shows that when notochordal cells were cultured for 3 days, the gene expression of cells cultured on PS control surfaces tended to be weaker than for those cultured on the various PPE:N surfaces. However, the difference was not significant. Its expression was subsequently maintained throughout the culture period, with no significant differences between the cells cultured on PS controls.

### Expression of pleiotrophin heparin binding factor (PTN)

**Figure 3:**
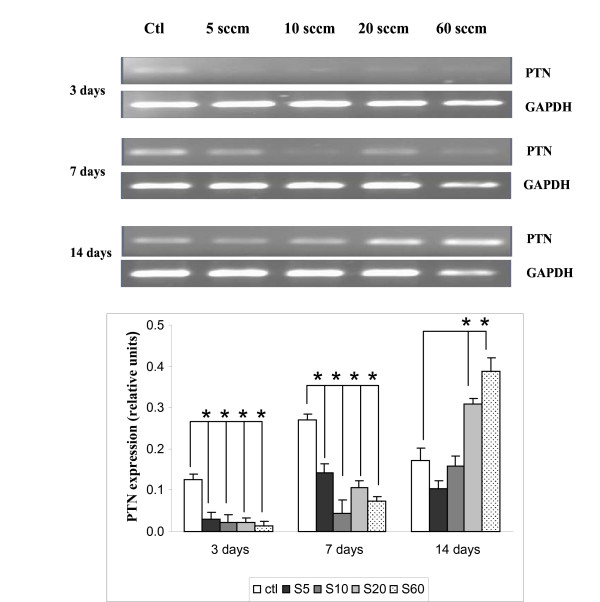
**PTN expression in dog notochordal cells cultured on five different substrates**. Dog notochordal cells were cultured for up to 14 days on PS control surface and four different PPE:N surfaces. GAPDH was used as housekeeping gene and served to normalize the results. Agarose gels are 0 standard error of these five experiments. * p < 0.05 vs. PS control.

Since PTN was recently discovered to be expressed in more than 10-fold higher concentration in the NP than in the AF [10], we next explored whether it is expressed in notochordal cells and whether PPE:N surfaces with different [N] values had any effect on its message. Figure 3 shows that PTN was weakly expressed in notochordal cells cultured on PS controls throughout the culture period, except for a transient increase on day 7. The PTN message was significantly down-regulated on PPE:N surfaces on days 3 and 7. On day 14, it was greatly up-regulated on S20 and S60 surfaces, but not on S5 and S10. These data indicate that PTN is regulated differently than VIM on these surfaces.

### Expression of matrix Gla protein (MGP)

**Figure 4:**
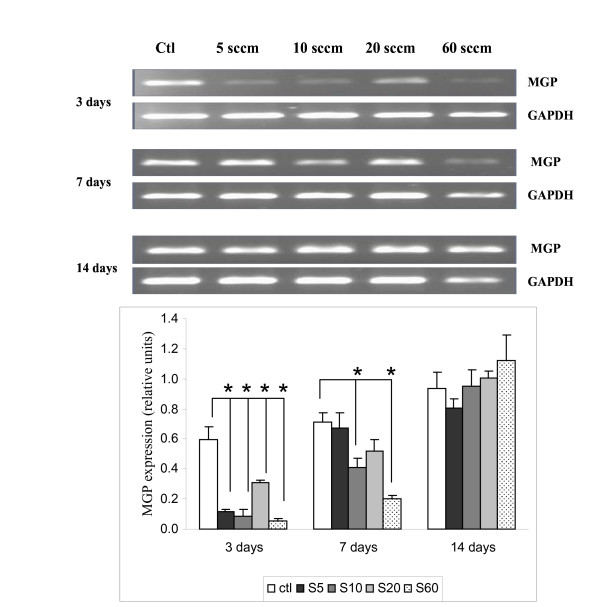
**MGP expression in dog notochordal cells cultured on five different substrates**. Dog notochordal cells were cultured for up to 14 days on PS control surface and four different PPE:N surfaces. GAPDH was used as housekeeping gene and served to normalize the results. Agarose gels are representative of 5 experiments. Quantitative results are the mean ± standard error of these five experiments. * p < 0.05 vs. PS control.

Figure 4 shows that the MGP message was consistently expressed on PS control dishes, increasing slightly with time from day 3 to day 14. In contrast, its expression was decreased when cultured on all four PPE:N coatings on day 3. On day 7, expression of this gene was still suppressed on S10, S20, and S60 surfaces, but no significant difference was observed between PS control and S5 PPE:N. However, on day 14, a slight decrease of MGP expression was observed on S5 while an increase was observed on S60.

### Expression of cartilage oligomeric matrix protein (COMP)

**Figure 5:**
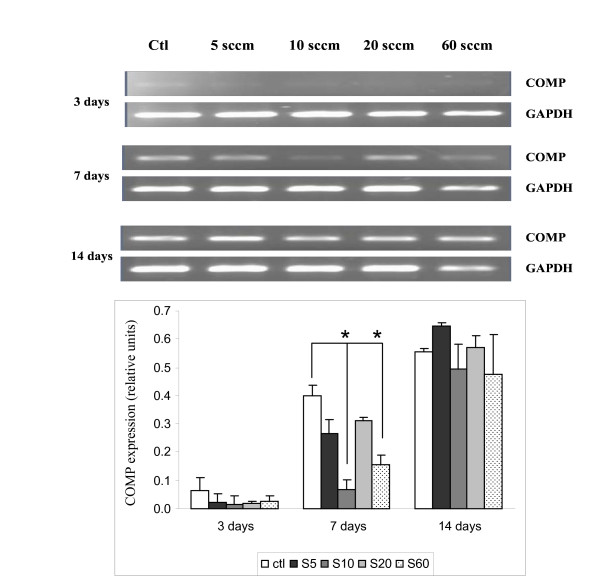
**COMP expression in dog notochordal cells cultured on five different substrates**. Dog notochordal cells were cultured for up to 14 days on PS control surface and four different PPE:N surfaces. GAPDH was used as housekeeping gene and served to normalize the results. Agarose gels are representative of 5 experiments. Quantitative results are the mean ± standard error of these five experiments. * p < 0.05 vs. PS control.

The results of RT-PCR analyses of COMP expression are shown in Figure 5. After culturing the cells for 3 days on PS control and on all four PPE:N surfaces, notochordal cells showed only very weak COMP messages. However, its expression increased progressively throughout the culture period up to 14 days. Also, its expression was lower on the PPE:N surfaces on day 7, indicating sensitivity towards [N] values. Finally, there was no significant difference of the expression of COMP on day 14 for all the surfaces (including the PS control) we studied.

### Expression of keratin 18 (KRT 18)

**Figure 6:**
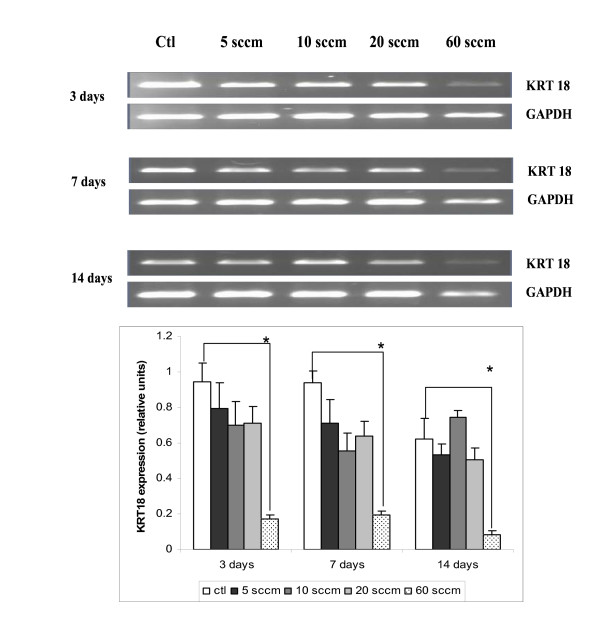
**KRT18 expression in dog notochordal cells cultured on five different substrates**. Dog notochordal cells were cultured for up to 14 days on PS control surface and four different PPE:N surfaces. GAPDH was used as housekeeping gene and served to normalize the results. Agarose gels are representative of 5 experiments. Quantitative results are the mean ± standard error of these five experiments. * p < 0.05 vs. PS control.

For keratin 19 (KRT 19), no messages were observed with primers, neither from the predicted dog gene sequence, nor from bovine sequences. It is important to note that the genome of dogs has not been studied as thoroughly as that of humans and bovines, and attempts to design primers according to the predicted sequence were to no avail. We therefore studied the expression of KRT 18, which was quite similar in notochordal cells cultured on PS controls and on S5, S10, and S20 PPE:N surfaces (Figure 6). However, its expression was significantly lower on S60 throughout the 14-day culture period. This suggests that a "critical" [N] value may exist for the expression of KRT 18, between 18.0 and 25.1% (see Table 1 and [9]).

### Expression of aggrecan (AGG)

**Figure 7:**
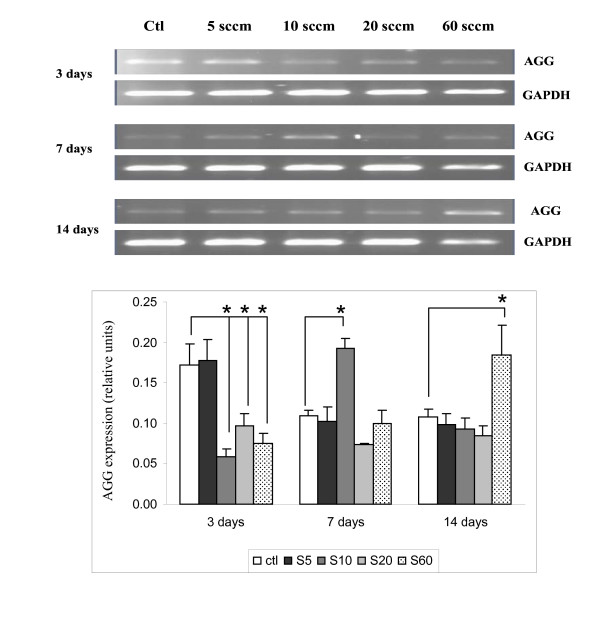
**AGG expression in dog notochordal cells cultured on five different substrates**. Dog notochordal cells were cultured for up to 14 days on PS control surface and four different PPE:N surfaces. GAPDH was used as housekeeping gene and served to normalize the results. Agarose gels are representative of 5 experiments. Quantitative results are the mean ± standard error of these five experiments. * p < 0.05 vs. PS control.

The expression of aggrecan was also consistently detectable in notochordal cells cultured on PS controls (Figure 7), although its expression decreased by day 7 and thereafter remained unchanged. Interestingly, its expression was quite unusual when cultured on PPE:N coatings, reaching maxima for S5, S10, and S60 on days 3, 7, and 14, respectively.

### Expression of type I collagen (COL1A2)

**Figure 8:**
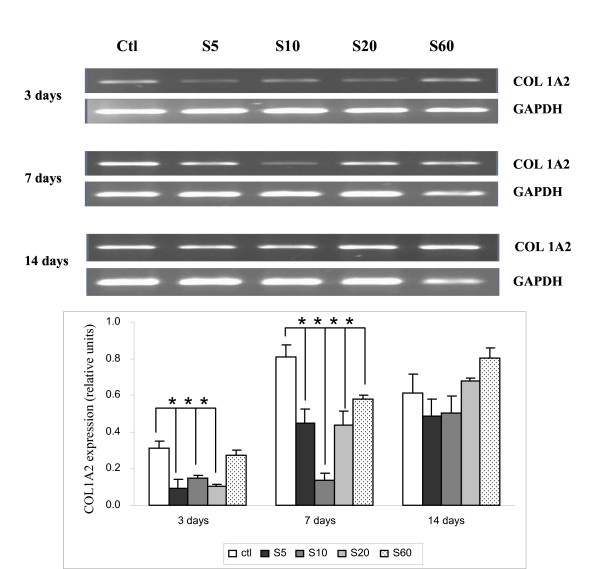
**COL1A2 expression in dog notochordal cells cultured on five different substrates**. Dog notochordal cells were cultured for up to 14 days on PS control surface and four different PPE:N surfaces. GAPDH was used as housekeeping gene and served to normalize the results. Agarose gels are representative of 5 experiments. Quantitative results are the mean ± standard error of these five experiments. * p < 0.05 vs. PS control.

Figure 8 shows that the COL1A2 message was consistently expressed on PS controls, enhanced somewhat on days 7 and 14. Its expression was decreased when cultured on S5, S10, and S20 PPE:N surfaces on day 3, compared to PS control. At day 14 all four PPE:N surfaces showed comparable results to that of the PS control.

### Expression of type II collagen (COL2A1)

**Figure 9:**
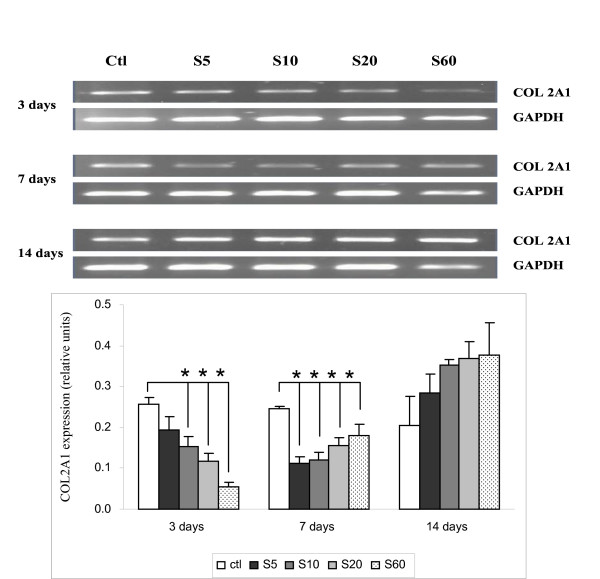
**COL2A1 expression in dog notochordal cells cultured on five different substrates**. Dog notochordal cells were cultured for up to 14 days on PS control surface and four different PPE:N surfaces. GAPDH was used as housekeeping gene and served to normalize the results. Agarose gels are representative of 5 experiments. Quantitative results are the mean ± standard error of these five experiments. * p < 0.05 vs. PS control.

Since COL2A1 is expressed in the NP, we explored its regulation in notochordal cells when cultured on PPE:N surfaces. Figure 9 shows that this gene was fairly weakly expressed in cells cultured on PS controls and on PPE:N surfaces. Its expression was maintained throughout the culture period on the former, but was lower on the latter four surfaces on days 3 and 7. Interestingly, on day 14, COL2A1 expression of cells cultured on all four PPE:N surfaces was up-regulated, and their dependences on days 7 and 14 appeared to vary inversely with [N], contrary to the [N]-dependence observed on day 3.

## Discussion

The immature nucleus pulposus (NP) is populated by cells of notochordal origin that are larger than NP cells, containing an extensive actin cytoskeletal network and numerous vacuoles [7,35]. They are the predominant cell type in the non-chondrodystrophoid dogs until approximately age 4 [41], and humans until roughly age 10 [42]. The precise biological function of these notochordal cells in the immature NP remains unclear. However, the loss of the notochordal cells from the NP in humans is associated with ageing and disc degeneration [7]. Although the molecular phenotype of notochordal cells is becoming more established [43,44] and their potential application in tissue engineering of the NP more apparent [7], little is known of their interaction with biomaterials. Recent studies suggest that the nature of scaffolds and substrates used for cell culture and in tissue engineering applications can influence cell behaviour [9,24,25,27-29,45].

The maturation of notochordal cells and the maintenance of their unique phenotypic profile are clearly complex processes. Examination in culture offers an opportunity to gain a better understanding of the different molecular changes and regulatory mechanisms that are involved in their disappearance with ageing. The availability in our group of PPE:N surfaces with very different [N] values [9] enables us to study the interactions of disc cells with a wide variety of different biocompatible substrates. As the chemically-bound N-functionalities on these surfaces can be controlled and reproduced at will, the role of synthetic polymeric material surfaces in regulating the response of cells to subtle changes in surface chemistry is now becoming amenable to understanding [9,29]. In our previous studies, it has been shown that gene expression can be specifically affected by different PPE:N surfaces in human mesenchymal cells (MSCs) [28,29]. The present study has provided additional new evidence that the chemistries of culture surfaces can affect specific gene expression profiles of notochordal cells from dogs. Indeed, we have shown that VIM, PTN, MGP, COMP, KRT 18, AGG and collagens I and II are expressed by dog notochordal cells and that their expression was affected by PPE:N, though VIM was affected to a lower degree. Results from MSCs [28,29] and notochordal cells (present study) suggest that the effect of [N] on gene expression may vary depending on the cell types under study. However, this remains to be investigated.

From the foregoing, it is therefore evident that, in dog notochordal cells, gene expression was markedly affected by the chemistries of the surfaces and the culture time. For the different PPE:N surfaces, the concentrations of [N] and [O] chemical functionalities at the surfaces varied as indicated in Table 1. All these changes presumably affected the interactions between the surfaces and the adhering notochordal cells. In other words, the interactions between the materials surfaces and notochordal cells result from a complex interplay of several different factors, in which the chemistries of the culture surfaces plays a primary role.

Vimentin, a major structural component of intermediate filaments in many types of cells, plays an important role in cell functions such as contractility, migration, and proliferation [46]. Vimentin (VIM)-positive cells were observed in IVDs [47]. Regional variations in the organization of the actin and VIM cytoskeletal networks were reported across all regions of the annulus [48]. Our current findings of VIM expression confirms and extends these earlier observations. It is worthy of note that similar VIM expression profiles were observed for all five culture surfaces examined, including PS controls; in fact, the surface chemistries and culture times had no significant effects on VIM expression. KRT 18 is a member of the subgroup of intermediate filament proteins and is important for the integrity of the intermediate filament network [49]. Its expression remained also quite stable when the notochordal cells were cultured on S5, S10, S20 and PS control surfaces, suggesting that the intermediate filament network of dog notochordal cells is not significantly affected by [N]. However, its expression was significantly down-regulated on S60 surfaces, suggesting differences in the regulation of VIM and KRT 18 expression in regards to [N]. The effect of [N] on other keratins remains to be investigated. Similar down-regulations of PTN, a secreted heparin binding cytokine with unusual and diverse functional activities largely in support of differentiation during development [50], MGP, an extracellular matrix protein probably involved in the prevention of ectopic calcification [51], and COMP, a non-collagenous protein of the ECM that is released during cartilage degradation [52], were also evident after 3 days of culture. However, after 14 days there was no significant down-regulation of PTN and MGP genes on S20 and S60 surfaces; indeed, PTN expression was even significantly up-regulated. COMP expression was also up-regulated but only after 7 days. Earlier studies by Ishii *et al*. [53] showed that COMP is expressed at higher levels in rat lumbar IVD than in its counterpart from the tails; they furthermore showed that within the IVD, COMP had greater expression in the AF than in the NP region, suggesting that it may play a role in the normal structure of IVD. Taken together, these results clearly demonstrate the time- and gene-specific effect of [N] on dog notochordal cells.

In regards to proteoglycans, it has been previously shown that NP cells co-cultured with notochordal cells exhibited an increase in aggrecan (AGG) synthesis [8]. Interestingly, the expression of AGG in purified notochordal cells seems very low, suggesting that the observed increase mentioned above may be the result of soluble factor(s) produced by notochordal cells. Thus, one might envisage embedding NP cells with notochordal cells, so that the latter can supply soluble factor(s) vital for enhanced matrix synthesis in tissue engineering applications. Studies of the human NP cell line indicates that they may become an alternative cell source for cell transplantation therapy in the treatment of IVD degeneration [54]. For collagens, the expression of COL1A2 and COL2A1 were significantly different with down-regulation of COL1A2 at day 3 on PPE:N surfaces and up-regulation after day 7, while COL2A1 was fairly weakly expressed throughout the culture period, in agreement with differences in the regulation of these genes in dog notochordal cells.

In previous studies, we have also examined the effects of different materials' surfaces on gene expression in human MSCs [27-29] and in foetal bovine NP cells (personal unpublished results). Taken together, it is evident that the interactions between polymeric surfaces and mammalian cells have been given much new impetus. Of course, given the limitations in available resources and in prevailing circumstances surrounding research in this field, we were obliged to study cells from different mammalian species. The effects of the different materials' surfaces on protein expression will likely give additional powerful support for the application of PPE:N-like scaffolds in tissue engineering.

## Conclusion

The present study has shown that notochordal cells from dogs can attach to and grow on PPE:N surfaces. Analysis of the expression of different genes in dog notochordal cells cultured on these different N-functionalized surfaces indicates that cellular behaviour is gene-specific and time-dependent. Further studies are required to better understand the roles of specific surface functionalities on the cells' receptor sites, and their effects on cellular phenotypes.

## Competing interests

The author(s) declare that they have no competing interests.

## Authors' contributions

FM designed the experiment and drafted the manuscript. HTW cultured the cells and designed the primers and analyzed the gene expression with RT-PCR. AP cultured the cells and structured the draft manuscript. PLGL prepared the PPE:N surfaces. CJH isolated the cells from dog lumbar spine. JAO worked on the statistical analyses. JA supervised the process of biological experiment. MRW designed the PPE:N surfaces and supervised the communication between the different groups. All authors read and approved the final manuscript.
